# A rare rib lesion due to parosteal osteosarcoma: a case report

**DOI:** 10.1186/s13256-018-1958-7

**Published:** 2019-01-23

**Authors:** Halil Tozum, Ahmet Nadir Aydemir, Murat Demiroglu, Korhan Ozkan, Ayse Bahar Ceyran, Bulent Kılıc

**Affiliations:** 1 Department of Thoracic Surgery, Istanbul Medeniyet University, Istanbul, Turkey; 2 Department of Orthopedics, Pamukkale Universty, Faculty of Medicine, Kınıklı, 20070 Denizli, Turkey; 3 Department of Orthopedics, Istanbul Medeniyet University, Istanbul, Turkey; 4 Department of Pathology, Istanbul Medeniyet University, Istanbul, Turkey; 5 Department of Physiotherapy and Rehabilitation, Istanbul Gelısım University, Istanbul, Turkey

**Keywords:** Parosteal osteosarcoma, Chest wall reconstruction

## Abstract

**Introduction:**

Masses which develop on the surface of the rib bones are rare. The differential diagnosis includes benign and malignant lesions.

**Case presentation:**

A 23-year-old European woman presented at an out-patient clinic with a 9-month history of a painless swelling on the right posterolateral side of her chest wall. The case reported here is of a very rarely seen parosteal osteosarcoma of the rib that was treated with wide resection and chest wall reconstruction. There was no evidence of local recurrence or distal metastasis after a 1-year follow-up.

**Conclusion:**

Parosteal osteosarcoma is a locally aggressive malignant tumor, and resection with a wide margin is the most appropriate treatment. Correct diagnosis of parosteal osteosarcoma is challenging for an orthopedic surgeon. Although rare, in the differential diagnosis of lesions located on the ribs, parosteal osteosarcoma should be considered and a systematic diagnostic approach should be taken.

## Introduction

Osteosarcomas (OSs) are the most frequent primary malignant bone tumor in which neoplastic cells produce osteoids and they usually develop in an intramedullary location [1]. Group OSs which originate from the outer surface of the cortex are known as surface OSs [2].

Parosteal OSs are the most common type of surface OSs and comprise approximately 4% of all OSs [3]. Parosteal OSs develop from the outer fibrous layer of the periosteum and are generally seen around the knee and proximal humerus.

Parosteal OSs are usually well-differentiated low-grade lesions with a better prognosis compared to conventional OSs [4]. The case here is a patient with a parosteal OS at a very rare location: arising from the rib. Parosteal OS is extremely rare in flat bones and there have been only a few cases in the English language literature reported as primary parosteal OS of the ribs [5–7].

## Case presentation

A 23-year-old European woman who was working as a secretary, presented at an out-patient clinic with a 9-month history of a painless swelling on the right posterolateral side of her chest wall. She did not recall any trauma that may be associated with this condition neither did she have a history of genetic disease or cancer. A physical examination revealed a hard, painless mass at the posteroinferior and lateral thoracic region. The results of her laboratory tests were all within normal limits.

**Fig. 1:**
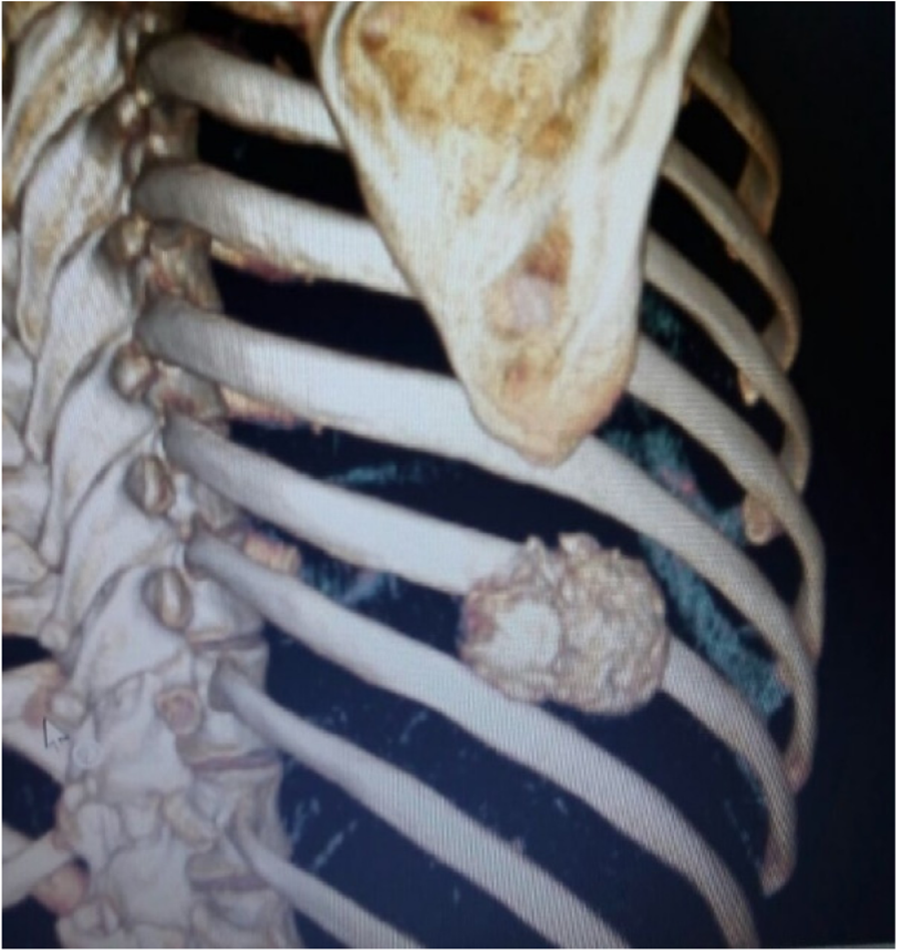
Preoperative three-dimensional computed tomography reconstruction of the chest

On a plain chest radiograph, an area of calcified opacity was observed at the ninth rib, with no destruction of the underlying bone. Computed tomography (CT) demonstrated a mass of 6 cm × 5 cm × 2.5 cm in size arising from the ninth rib (Fig. 1). There was no evidence of cortical destruction or medullary involvement of the rib. Whole-body scintigraphy and CT did not show any skip or lung metastases.

After these examinations, an incisional biopsy was performed. Histopathologic examination revealed fibroblastic and osteoblastic cells with mild nuclear atypia and pleomorphism which was consistent with parosteal OS.

**Fig. 2:**
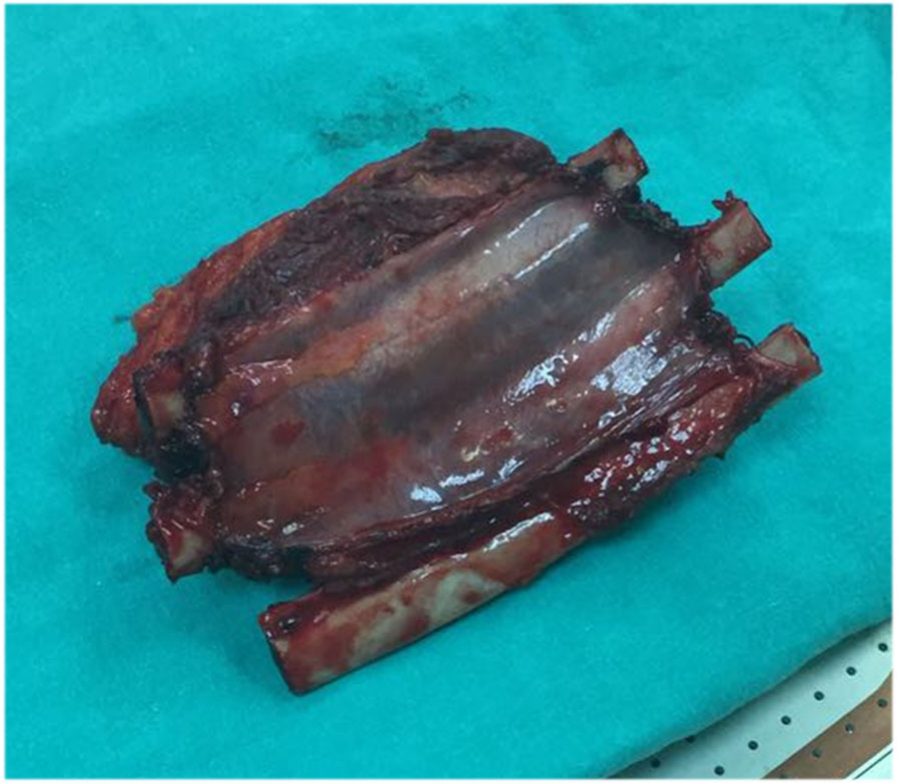
Gross specimen of the mass shows three ribs and underlying pleura

**Fig. 3:**
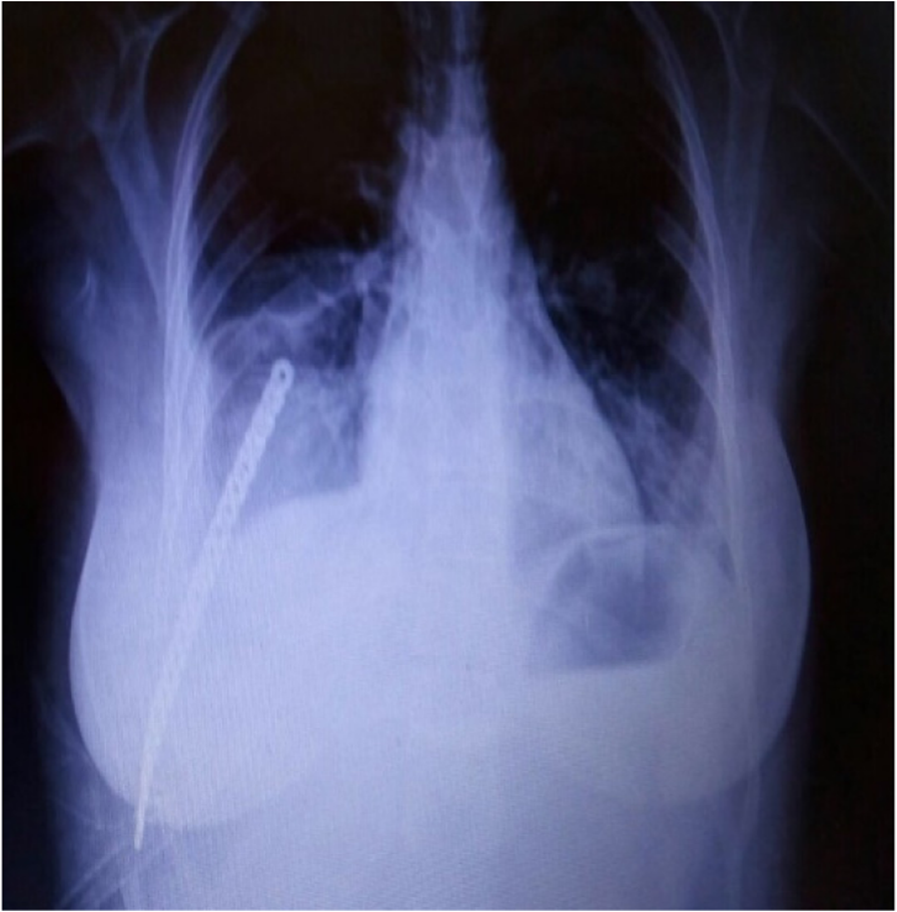
Postoperative chest radiograph. Reconstruction made with collagen mesh and a locked plate

She was informed and a wide segmental resection was applied to her eighth, ninth, and tenth ribs with the involvement of parietal pleura (Fig. 2). Afterward, chest wall reconstruction was made using collagen mesh and low-profile locked plate for the prevention of flail chest (Fig. 3).

On gross examination, the lesion was found to be attached to the outer surface of her ninth rib measuring 7 cm × 3 cm × 5 cm. Histopathological evaluation of the resected specimen confirmed it to be parosteal OS. Our patient had no chemotherapy and throughout a 1-year follow-up, there was no evidence of local recurrence or distal metastasis. Informed consent was obtained from our patient before this report.

## Discussion

Parosteal OSs are uncommon bone tumors and comprise only 4% of all OSs [3]. While the radiological features of parosteal sarcoma of the long bones are well described, they may be difficult to detect on a simple chest radiograph. In the current case, a dense mass could be seen but it is imperative to perform a meticulous diagnostic workup as it can be confused with other benign and malignant lesions. A CT scan is one of the important diagnostic imaging methods as it can accurately delineate the extent of the tumor invasion and cortical integrity of the underlying bone. Magnetic resonance imaging (MRI) is another option to support a CT scan and give information about soft tissue, medullary involvement, and the differential diagnosis.

The differential diagnosis of parosteal OS from other bone-forming benign and malign neoplasms is yet another complexity [8]. Osteochondroma is usually a pedunculated or rarely a sessile mass covered by a thin cartilage cap usually less than 2 cm. As osteochondroma and parosteal OS share some common clinical and morphologic features, differential diagnosis of parosteal OS from osteochondroma is usually the most challenging. Both of them arise from the cortex and have a similar location and age characteristics. The chondrocytes of parosteal OS usually show more pleomorphism and occasional binucleation and a lack of regular columnar arrangement and polarity [9]. Myositis ossificans may be grossly similar to parosteal OS, radiographically, however, the ossification pattern of parosteal OS is generally centrally located whereas the ossification pattern of myositis ossificans is peripherally located [10]. In addition, lesions of parosteal OS on MRI are more hypointense both in T1-weighted and T2-weighted images [10]. A high-grade surface OS is another entity that may be confused with parosteal OS. A differential diagnosis is usually made by histopathological examination. Spindle cells with marked nuclear atypia and lacelike osteoid production, as seen in conventional OS, are usually evident in high-grade surface OS [11].

The medical history of the patient is also very important because fracture callus and ossifying hematoma can easily simulate bone tumors. So, clinical, radiologic, and pathologic assessments of the lesions are paramount evidence for the parosteal OS differential diagnosis.

Parosteal OS is a locally aggressive malignant tumor, and resection with a wide margin is the most appropriate treatment. In the current case, the posterolateral part of our patient’s ninth rib was resected en bloc with her eighth and tenth ribs. Her chest wall was then reconstructed to avoid future pulmonary compromise. In general, chest wall reconstruction is recommended for defects > 5 cm or when more than two ribs are resected to avoid flail chest. In the current case, the chest wall defect was > 10 cm and three ribs were resected with intercostal muscles and parietal pleura to achieve a clear margin. The chest wall reconstruction was applied with a collagen mesh and locked plate and no complications were observed during follow-up.

## Conclusion

A rare case of parosteal OS located on a rib is presented here. Although rare, for the differential diagnosis of lesions located on the ribs, parosteal OS should be considered and a systematic diagnostic approach should be taken.

## Abbreviations

CT: Computed tomography
MRI: Magnetic resonance imaging
OS: Osteosarcoma
